# Clinical Significance of Altered Vascular Morphology and Function in Normotension

**DOI:** 10.1007/s11906-023-01251-7

**Published:** 2023-07-01

**Authors:** A. Triantafyllou, P. Anyfanti, N. Koletsos, A. Malliora, S. Lamprou, K. Dipla, E. Gkaliagkousi

**Affiliations:** 1 Third Department of Internal Medicine, Papageorgiou General Hospital, Aristotle University of Thessaloniki, 56429 Thessaloniki, Greece; 2 Second Medical Department, Hippokration Hospital, Aristotle University of Thessaloniki, 54642 Thessaloniki, Greece; 3 Physiology & Biochemistry Laboratory, Department of Sport Sciences at Serres, Aristotle University of Thessaloniki, 62100 Serres, Greece

**Keywords:** Arterial stiffness, Atherosclerosis, Retinopathy, Albuminuria, Capillary rarefaction, Normotension, Microcirculation, Macrocirculation

## Abstract

**Purpose of Review:**

To review current literature examining the presence of subclinical micro- and macrovascular alterations in normotensive individuals and their clinical significance in terms of hypertension prediction. Emphasis is placed on alterations that can be detected in peripheral vascular beds using non-invasive, easily applicable methodology, as these are in general easier to capture and evaluate in clinical practice compared to more complex invasive or functional tests.

**Recent Findings:**

Arterial stiffness, increased carotid intima-media thickness, and altered retinal microvascular diameters predict the progression from the normotensive to the hypertensive state. By contrast, there is substantial lack of relevant prospective studies for skin microvascular alterations. Although conclusions regarding causality cannot be safely deduced from available studies, detection of morphological and functional vascular alterations in normotensive individuals emerges as a sensitive indicator of progression to hypertension and hence increased CVD risk.

**Summary:**

An increasing amount of evidence suggests that early detection of subclinical micro- and macrovascular alterations would be clinically useful for the early identification of individuals at high risk for future hypertension onset. Methodological issues and gaps in knowledge need to be addressed before detection of such changes could guide the development of strategies to prevent new-onset hypertension in normotensive individuals.

## Introduction

Cardiovascular disease (CVD) remains the major cause of mortality globally among adults aged 35–70 years [1]. It has been estimated that over 70% of CVD cases and deaths can be attributed to modifiable risk factors, with hypertension exerting the most extensive effects globally. However, marked reductions in deaths from CVDs have been observed in the last couple of decades especially in high-income countries, related to recent advances in prevention and treatment of non-communicable diseases [2, 3]. Comprehensive understanding of the clinical course of CVDs has mitigated the development and implementation of health care policies aiming to control common modifiable CVD risk factors and improve existing preventive treatments.

Endothelial dysfunction is perceived as the earliest precursor of CVD, triggering the cascade of accelerated atherosclerosis, subclinical vascular injury, and subsequently clinically evident CVD manifestations [4]. In this pathophysiological sequel, subclinical vascular damage represents the first alarming sign of probable future CVD that can be easily identified in vivo at a clinical level. As a leading cause of CVD, hypertension affects primarily the vasculature. The term “hypertension-modified organ damage (HMOD)” has been introduced to describe the complications of hypertensive vascular disease, which are listed under the pathologic heading of either cardiac hypertrophy, arteriosclerosis, or arteriolosclerosis [5, 6]. Detection of asymptomatic HMOD has been made easy and affordable through the implementation of appropriate non-invasive tools and is warranted in hypertensive individuals as it may affect therapeutic strategies [5].

However, the wide applicability of such markers of vasculopathy has revealed the presence of micro- and macrovascular alterations in a considerable portion of individuals with normal blood pressure levels as well. Nevertheless, the clinical importance of such changes in normotensive individuals has received much less attention compared to other high CVD risk populations, and their presence is frequently overlooked in clinical practice as many physicians remain unaware of their prognostic significance.

**Fig. 1:**
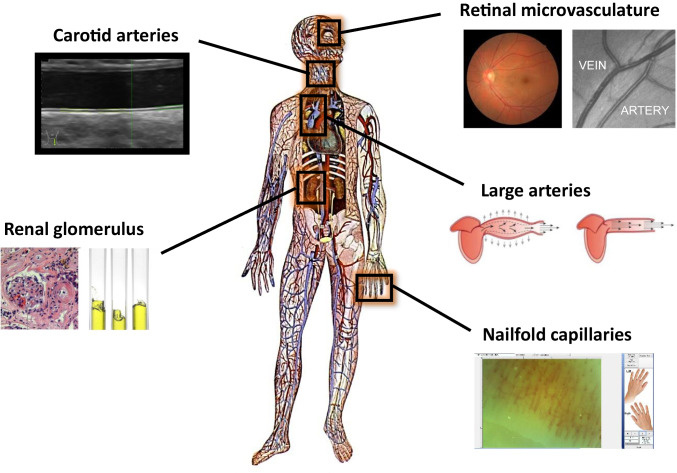
Easily accessible vascular beds for the non-interventional, in vivo study of micro- and macrocirculation. Arterial stiffness and carotid intima-media thickness, secondary to changes in the mechanical and anatomical properties of the arterial wall, are the most widely applied, well-established markers of macroangiopathy. The retina, kidney, and the skin may be used as windows to the heart for the study of microcirculation. While measurement of urinary albumin excretion has long been used as an early indicator of generalized microvascular impairment, methodological advances facilitate the extraction of valuable information on microcirculation from images capturing the retinal microvasculature and the skin capillary network

Keeping in mind the pathophysiological and prognostic implementations of early vascular changes, the present review aims to summarize subclinical vascular alterations and their clinical significance in normotensive individuals. We will focus on changes that can be detected non-invasively in peripheral vascular beds as presented in Fig. 1, as these are in general easier to capture in real-world clinical practice compared to more complex invasive or functional tests. Specifically, we will present prospective studies providing evidence of such alterations in normotensive individuals detected by means of non-invasive, easily applicable methodology in the large vessels (aorta, carotid arteries) and the microvasculature (retina, nephrons, dermal capillary network). Their clinical significance will be discussed, with emphasis placed on their prognostic value in terms of hypertension prediction. To this end, a PubMed search was performed to identify relevant articles published in English, using the following medical terms: “arterial stiffness”; “carotid atherosclerosis”; “microalbuminuria”; “retinopathy”; and “capillary rarefaction”.

## Arterial Stiffness

Large artery stiffening corresponds to a degradation of the anatomical properties of the arterial wall resulting in a decrease of the high elastin to collagen ratio under the impact of various systemic diseases, particularly those primarily affecting the cardiovascular system [7]. Evaluation of arterial stiffness has been established as a reliable marker of biological age and has been acknowledged as a surrogate marker of CVD. There is indisputable evidence from longitudinal studies in tenths of thousands of individuals to support the incremental prognostic value of arterial stiffness in terms of CVD morbidity and mortality, especially in high-risk groups (coronary artery disease, renal disease, hypertension) but also among low-risk subjects (general population) [8]. Considering the plethora of data, arterial stiffness represents one of the most studied manifestations of early vascular damage in normotensive individuals.

Main prospective studies providing evidence of the prognostic value of increased arterial stiffness using the gold-standard pulse wave velocity (PWV) for future development of hypertension are presented in Table 1. As early as in 1999, the ARIC (Atherosclerosis Risk in Communities Study) [9] showed that in a cohort of 6992 normotensive men and women, arterial elasticity (as estimated by adjusted arterial diameter change, Peterson’s and Young’s elastic modulus, and *β* stiffness index from ultrasound) was an independent predictor for the development of hypertension over a 6-year follow-up period. A decrease in arterial elasticity by one standard deviation was associated with a 15% increased risk of hypertension, and this association was independent of established risk factors for hypertension and the level of baseline blood pressure. In the Baltimore Longitudinal Study of Aging, increased PWV was an independent predictor of incident hypertension [10]. In particular, there was a 10% increased risk for developing hypertension for every 1 m/s increase in carotid-femoral PWV (cfPWV), suggesting that PWV could help identify high-risk normotensive individuals who could be eligible for interventions aiming at preventing or delaying the onset of hypertension. The Framingham Offspring Study yielded similar results [11]. In a multivariate adjusted regression model, cfPWV (OR: 1.3, 95% CI 1.0–1.6, *p* = 0.04), augmentation index (OR: 1.7, 95% CI 1.4–2.0, *p* < 0.001), and forward wave amplitude (OR: 1.6, 95% CI 1.3–2.0, *p* < 0.001) were independently related to incident hypertension, further supporting the notion that vascular stiffness is a precursor rather than the result of hypertension.

**Table 1:** Prospective studies evaluating the prognostic value of PWV for future development of hypertension

Study	Year published	PWV method	Study population	Number of participants	Time of follow-up	Results
Yambe et al	2007	Brachial-ankle	Employees of a single large construction company	1758 non-hypertensive men (475 with high normal BP)	2000–2004	PWV in the highest quartile was significantly predictive of progression to hypertension in individuals with high normal BP
Najjar et al	2008	Carotid-femoral	Baltimore Longitudinal Study of Aging	306 non-hypertensives	4.9 ± 2.5 years	PWV was an independent predictor of incident hypertension
Tomiyama et al	2009	Brachial-ankle	Employees of a single large construction company (company, headquarters, and offices)	777 prehypertensive men	2003–2006	PWV and BMI are not powerful but significantly independent markers to identify men with prehypertension at high risk for hypertension
Satoh et al	2011	Brachial-ankle	Male employees of a single local government agency	2278 non-hypertensives	3-year follow-up	PWV is a significant and independent predictor of incident hypertension
Takase et al	2011	Brachial-ankle	Individuals undergoing yearly health check-up in Japan	2496 non-hypertensives	733 ± 360 days	PWV is a significant independent predictor of both longitudinal increases in BP and new onset of hypertension
Kaess et al	2012	Carotid-femoral	Framingham offspring cohort	1048 non-hypertensives	1998–2001 2005–2008?	Higher PWV, AIx, and FWA were associated to incident hypertension
Zheng et al	2015	Brachial-ankle	Asymptomatic Polyvascular Abnormalities Community Study	2153 non-hypertensives	An average of 27 months	PWV is an independent predictor of BP progression and incident hypertension
Kim et al	2016	Brachial-ankle	Korean Genome Epidemiology Study	1785 non-hypertensives	4-year follow-up	PWV independently predicts incident hypertension
Koivistoinen et al	2018	From aortic arch to popliteal artery	Cardiovascular Risk in Young Finns Study	1183 non-hypertensives	2007–2011	PWV directly and independently predicts an increase in BP and the development of hypertension
Lee et al	2019	Brachial-ankle	Kangbuk Samsung Healthy Study	10,360 non-hypertensives	2.17 (1.74–3.63) years	PWV independently associated with incident hypertension Women were at higher risk
Jiang et al	2020	Brachial-ankle	Shougang cohort study	1839 non-hypertensives	2.4 (2.3–2.4) years	PWV independently and gradually predicted the risk of hypertension and BP progression, modified by the level of SBP at baseline

Similar results were found in studies using the brachial-ankle pulse wave velocity (baPWV). In more details, in a Korean population of 10,360 young and middle-aged healthy adults, it was found that baPWV was independently associated with incident hypertension, with a stronger risk among females compared to the male population of the study [12]. The independent association between baPWV and incident hypertension was also observed in smaller studies including normotensives [13, 14••, 15–17]. These findings were also confirmed in a Japanese study of 2496 participants (27–84 years) with follow-up of 5215 person-years, in whom baseline value of baPWV was associated not only with new onset of hypertension after adjustment for known risk factors but also with a longitudinal increase in blood pressure in multiple regression analysis [18]. Implementation of baPWV in individuals with high normal blood pressure has further confirmed its predictive value for progression to hypertension, with a 3-year observational period of 524 non-hypertensive individuals showing that assessment of arterial stiffness with baPWV was more reliable for predicting the progression to hypertension in cases of prehypertension compared to individuals with normal and optimal blood pressure [19]. In addition, several studies have demonstrated a positive yet weak association of PWV with future development of hypertension in prehypertensive males [19, 20] and normotensive populations of different race included in the Multi-Ethnic Study of Atherosclerosis [21].

Apart from the gold-standard measurement of PWV, implementation of other markers of arterial stiffness has been suggested to predict the progression to hypertension. In a population of 2512 non-hypertensive individuals, those with aortic inelasticity (as indicated by aortic strain, distensibility, and stiffness index *β* assessed with echocardiography) were more likely to develop hypertension after 4 years of follow-up. Notably, arterial stiffness remained significantly associated with incident hypertension after adjustment to classical CVD risk factors, both in men and women and in young and old populations [22]. Recently, the cardio-ankle vascular index (CAVI), another marker of arterial stiffness, has also been associated with increased incidence of hypertension. Specifically, a Japanese study in 34,649 normotensives without any CVD showed that increased baseline CAVI elevates the risk of hypertension (HR 1.32 [1.25–1.39] 95% CI) [23].

## Carotid Atherosclerosis

Atherosclerosis is a primary pathophysiological process that typically remains silent for several years until the development of clinically evident CVD manifestations [24]. The atherosclerotic burden in apparently healthy people can be easily and non-invasively detected in the carotid arteries using ultrasonography, as a reliable indicator of systemic atherosclerosis. While advanced stages of atherosclerosis include carotid plaque, stenosis, and occlusion, carotid intima-media thickness (cIMT) has been established as a robust marker of subclinical atherosclerosis, especially with values exceeding 1.0 mm which are generally considered as abnormal [25]. Increased cIMT is associated with common CVD risk factors such as current smoking, diabetes, and hypertension and with both prevalent and incident CVD and has been widely used in outcome trials as a surrogate or predictor of CVD outcomes [26•, 27].

Several studies have shown that cIMT independently predicts incident hypertension. In the Multi-Ethnic Study of Atherosclerosis [21], 2512 normotensive participants, with a mean age of 58 years, underwent a high-resolution B-mode ultrasound, in which the cIMT of common and internal carotid arteries was measured. After a follow-up of 4.3 years on average, a significant positive association between increased maximum common cIMT and new-onset hypertension was found. Similar results emerged from a 4-year follow-up study in 1785 non-hypertensive adults, aged 40–69 years [16]. In line with the aforementioned results, another study in 672 normotensives [28] showed that both the average (OR = 1.83, 95% CI: 1.46–2.29, *p* < 0.0001) and maximum (OR = 1.68, 95% CI: 1.38–2.05, *p* < 0.0001) values of common carotid artery IMT significantly correlated with incident hypertension. Furthermore, an observational study involving 867 normotensive participants, aged 25 years or older, with a 3-year median follow-up approximately indicated that increased carotid IMT elevated the risk of hypertension by 63%, although a causal relationship between an increased cIMT and the development of hypertension was not established [29]. Nevertheless, findings from two other studies showed that healthy offspring of hypertensives presented increased cIMT compared to healthy decedents of normotensives [30, 31], reinforcing the hypothesis that increased cIMT, even within the normal range, may increase the risk of hypertension.

## Urinary Albumin Excretion

Moderately increased albuminuria (MIA), the new terminology for microalbuminuria, defined as urinary albumin excretion between 30 and 300 mg/24 h or urinary albumin/creatinine ratio (UACR) between 30 and 300 mg/g, is a well-recognized non-invasive index of renal microcirculation [32, 33]. The prevalence of MIA in the general population was found to be 7.8% [34]. Increased urinary albumin excretion has been acknowledged as a powerful and independent predictor of prognosis in several disease states, such as diabetes, hypertension [35], and heart failure [36], and has been consistently used as a surrogate endpoint in therapeutic trials [37] for both cardiovascular and renal outcomes. In addition, albuminuria and estimated glomerular filtration rate have been correlated with all-cause and CVD mortality in the general population according to a meta-analysis in 105,872 participants [38].

There is evidence that the presence of MIA was associated with an increased risk for developing hypertension. More specifically, results from the Framingham Offspring Study showed that UACR, even below the threshold for microalbuminuria, was associated with an increased risk for developing hypertension in non-diabetic and non-hypertensive individuals [39]. In this study, a total of 9 different biomarkers were simultaneously measured to conclude that urinary albumin excretion was one of the three markers (along with C-reactive protein and plasminogen activator inhibitor-1) that remained as significant predictors of future incidence of hypertension [39]. More recently, Zhang et al. found that MIA and hypertension are associated in a bidirectional way [40••]. In that longitudinal study, baseline microalbuminuria predicted the risk of incident hypertension (odds ratio = 1.75, *p* = 0.028), and baseline blood pressure also significantly predicted the risk of microalbuminuria (odds ratios = 1.27 and 1.21 for a per-SD increase in systolic and diastolic blood pressure, respectively). While elevated urinary albumin excretion was more likely to precede hypertension, conclusions regarding causality effects could not be provided.

It should be noted that not only MIA but even a slight increase in UACR, within the normal range, is a risk factor for incident hypertension as shown in several studies including normotensive [41–44] and healthy [45–48] populations. Specifically, in 2016, a 10-year follow-up study [41] involving 9102 normotensive individuals showed that increased UACR, even without exceeding the threshold of 30 mg/g, is an independent risk factor for hypertension (highest UACR quartile HR 1.95 [95% CI 1.51, 2.53]; trend across UACR quartiles *p* < 0.001). Similarly, the Takahata study [47] established a significant association between elevated UACR, even below the threshold of ≥ 30 mg/g, and incidence of hypertension (OR 2.35, 95% CI 1.28–4.46 for UACR 5–9.9 mg/g and OR 2.78, 95% CI 1.44–5.52 for UACR 10–29.9 vs. UACR 5 mg/g) in 412 participants, free of hypertension, diabetes, renal insufficiency, microalbuminuria (UACR ≥ 30 mg/g), and cardiovascular or renal disease.

Furthermore, MIA has been associated with incidence of CVD events and all-cause mortality in healthy individuals [49, 50]. Analysis of the Framingham study [39] data showed that the presence of microalbuminuria above the median, ≥ 3.9 μg/mg in men and ≥ 7.5 μg/mg in women, was associated with a threefold increased risk of CVD (adjusted HR 2.92, 95% CI 1.57 to 5.44, *p* < 0.001) and for a marginally insignificant increase in total mortality (adjusted HR 1.75, 95% CI 0.95 to 3.22, *p* = 0.08) compared to those with a lower-than-average UACR. Furthermore, a 6-year follow-up [49] in 1568 participants, without hypertension, diabetes, or CVD at baseline, suggested that increased UACR, even within the normal range, elevates the mortality risk (HR, 1.55; 95% CI, 1.10 to 2.20; *p* = 0.014), while UACR at or above the sex-specific median increases the risk of CVD by nearly 3, compared with those with UACR below the median [HR, 2.92; 95% CI, 1.57 to 5.44; *p* = 0.0007]. Finally, a study in 1318 participants, without a history of diabetes mellitus, hypertension, coronary artery disease, and chronic kidney disease, showed an association between UACR and coronary atherosclerosis, as detected by coronary artery computerized tomography [51].

## Retinal Microvascular Alterations

The retina provides an easily accessible window for the study of microcirculation [52]. While clinical fundoscopic evaluation remains the classical method, digital retinal photography can also be applied as a simple and reproducible method to diagnose hypertensive retinopathy. Advanced stages of hypertensive retinopathy have been long considered as an index of HMOD in other organs and systems, such as the kidney. However, the significance of early-stage alterations in the retinal microvasculature is being increasingly recognized in non-hypertensive individuals.

**Fig. 2:**
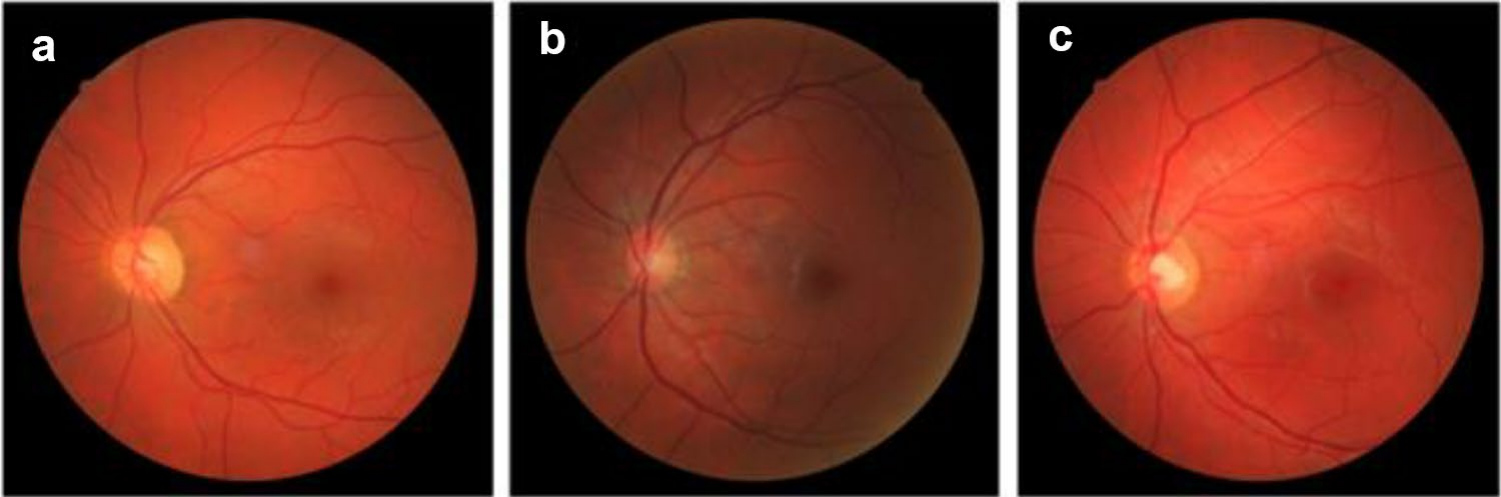
Subtle alterations of the retinal arteriolar and venular width can be calculated from digital retinal photography by use of appropriately designed software and have been associated with increased risk of future hypertension onset in multitudinal prospective studies. **a** Obtained from a 38-year-old normotensive male, arteriolar and venular widths are almost equal corresponding to arteriovenous ratio (AVR) of 1.04. **b** Obtained from a 32-year-old healthy normotensive female, retinal arteriolar narrowing is evident (AVR 0.69). These changes are remarkably similar to those observed in established hypertension, as shown in **c** from a 32-year-old hypertensive male (AVR 0.725). Figure obtained and provided by authors (ESH Excellent Center: Hypertension Division of the Third Department of Internal Medicine, Aristotle University of Thessaloniki, Papageorgiou General Hospital, Thessaloniki)

Qualitative fundus evaluation using traditional classification systems may reveal arteriosclerotic changes in the retinal microvasculature. For instance, signs of retinopathy assessed with the Scheie classification in fundus photographs were associated with future onset of hypertension in a huge population of 34,649 normotensive individuals without any CVD, after a mean follow-up period of 3.18 years [23]. However, qualitative evaluation is subjective and requires expertise. On the other hand, digital retinal photography enables the implementation of software to facilitate the quantitative assessment of morphological alterations, such as calculation of arteriolar and venular width, as presented in Fig. 2 [53, 54]. In more details, changes in the caliber of retinal arteries and veins can predict new onset of arterial hypertension. Results from a 10-year follow-up of 2451 normotensive individuals (Beaver Dam Study) showed that, regardless of classic CVD risk factors (age, smoking, lipid levels, diabetes, body mass index, and initial blood pressure levels), individuals with the lowest retinal arteriole-to-venule ratio (AVR) (1^st^ quadrant), compared with those with the highest (4^th^ quadrant), were 80% more likely to develop high blood pressure [55]. This relationship was also confirmed when the AVR was studied as a continuous variable, with a 30% increase in hypertension for each decrease of a standard deviation (SD: 0.07) of the AVR. In addition, the same study showed that the linear relationship of blood pressure values with arteriolar width and AVR was valid for healthy individuals and comprised an independent important prognostic factor in the development of hypertension.

Concordant results emerge from a 3-year follow-up of 5628 normotensives (49 to 73 years old) [56] and a 5-year follow-up of a normotensive Japanese population (Funagata study—1058 participants > 35 years old) [57], as well as from a 7-year follow-up of an older normotensive population (Rotterdam study—1900 participants ≥ 55 years old) [58]. According to these studies, narrowing of the retinal arteries in initially normotensive individuals was significantly associated with an increased incidence of hypertension in the future. In the Multi-Ethnic Study of Atherosclerosis [59] that included 2583 normotensive participants without a history of clinical CVD, not only narrower retinal arteries (referred to as central retinal artery equivalent) but also wider retinal veins (referred to as central retinal vein equivalent) were also identified as independent risk factors for future development of hypertension. Likewise, the meta-analysis by Ding et al. including six population-based studies showed that both narrowing of the retinal arterioles (OR per 20 μm difference in diameter 1.29, 95% CI 1.20–1.39) and venular widening (OR per 20 μm difference in diameter 1.14, 95% CI 1.06–1.23) were significant predictors of future onset of hypertension among 10,229 individuals without prevalent hypertension, diabetes, or CVD [60].

More recent studies have tested the efficacy of novel computerized approaches to predict of hypertension and CVD outcomes. Complementary machine learning-based assessment of the retinal vasculature with phenome-wide and genome-wide analyses was leveraged across 97,895 retinal fundus images from 54,813 UK Biobank participants. Vascular density and fractal dimension as a measure of vascular branching complexity were calculated using convolutional neural networks to segment the retinal microvasculature. Both were significantly associated with a higher risk for incident mortality, hypertension, congestive heart failure, renal failure, type 2 diabetes, sleep apnea, anemia, and multiple ocular conditions epidemiologically, while lower microvascular density was further associated with genetically higher risk for hypertension and diabetes [61••]. Though promising, the advantages of applying deep-learning techniques in the retina need to be counterbalanced over feasibility for incorporation into clinical screening procedures.

## Alterations of the Skin Microcirculation

The skin represents an easily accessible vascular bed, and as such, several techniques have been developed for the study of skin microcirculation. For instance, traditional laser Doppler techniques (laser Doppler imaging and laser Doppler flowmetry) that evaluate and quantify red cell flux in small areas of tissue, and more recently the laser speckle contrast imaging, have been applied to document altered microvascular reactivity in systemic diseases such as autoimmune rheumatic disorders and hypertension [62–64, 65•]. Nevertheless, these techniques have not yet been applied in initially normotensive individuals to evaluate prospectively their potential value in terms of CVD risk prediction.

**Fig. 3:**
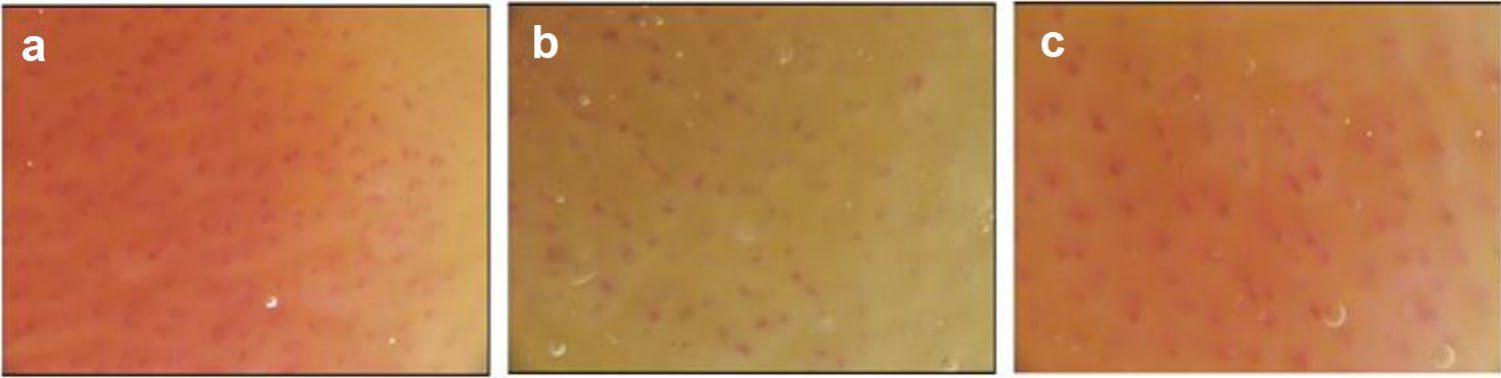
Nailfold capillaroscopy provides valuable information on capillary rarefaction as an index of peripheral microangiopathy. Skin capillary network appears dense in a 48-year-old normotensive female in Fig. 1a. By contrast, capillary rarefaction is evident in a 46-year-old normotensive male presented in Fig. 1b, despite the absence of established hypertension, as is the case in Fig. 1c (48-year-old hypertensive male). Figure obtained and provided by authors (ESH Excellent Center: Hypertension Division of the Third Department of Internal Medicine, Aristotle University of Thessaloniki, Papageorgiou General Hospital, Thessaloniki)

Capillaroscopy is another non-invasive technique that can visualize by use of a stereo microscope the capillary network of the studied organ, usually the skin of the dorsum of the nail of the upper extremities. Nailfold capillaroscopy has been primarily used in rheumatology for the diagnosis and monitoring of autoimmune rheumatic diseases. It can provide valuable information for multiple microcirculatory parameters, including the morphology and density of the capillary network, under the influence of various systemic diseases [66]. Using nailfold capillaroscopy, reduced number of skin capillaries can be detected, which is known as capillary rarefaction (Fig. 3) and represents a common alteration found in hypertensive patients [67], as well as in other high CVD risk populations [68].

Nevertheless, the predictive role of capillary rarefaction for the development of hypertension in normotensive individuals has not been investigated to date with prospective studies. Relevant data come from cross-sectional studies in patients with hypertension [69] and in healthy individuals with a family history of hypertension [70, 71], suggesting that capillary rarefaction may be preexisting and could be partly responsible for the increased peripheral resistance preceding the establishment of arterial hypertension itself. A proposed pathophysiological mechanism underlying capillary rarefaction among normotensives involves metabolic abnormalities. Serne and colleagues [72], using the euglycemic clamp, showed a significant correlation between insulin resistance and capillary density in normotensive individuals (*n* = 18). This correlation of insulin resistance, however, was not confirmed in a larger (*n* = 105) group of healthy individuals, using the HOMA index, an indirect and less accurate way of assessing insulin resistance compared to the euglycemic clamp and only fasting glucose correlated with capillary rarefaction [73]. Finally, a study in 66 normotensives examining the potential relationship between impaired metabolic profile and capillary rarefaction reported a significant association between high-density lipoprotein levels and capillary density [74].

## Gaps in Knowledge and Future Perspectives

Evaluation of arterial stiffness, carotid atherosclerosis, and retinal and skin microvascular changes is non-invasive, prompt, and feasible in routine clinical practice. Nevertheless, these measures are not routinely applied as part of the general check-up of healthy individuals. First, these methods necessitate expertise and relevant infrastructure, although once obtained consumables are not required. Second, with the exception of arterial stiffness measures and urine albumin excretion (UAE), methodological standardization remains the Achilles’ heel. This is particularly true for cIMT despite several decades of experience, which is therefore not recommended by current European Society of Cardiology guidelines on CVD prevention in clinical practice [75]. While several research centers or groups have developed software for the evaluation of retinal and skin microvascular alterations from digital fundus photographs and nailfold capillaroscopic images, respectively, the need for universal software and protocols emerges as extremely important. Third, the development of diagnostic thresholds remains troublesome especially for retinal and skin microvascular alterations. While cut-off levels have been developed for PWV, cIMT, and UAE to discriminate abnormal values, there is increasing awareness of the linear association with CVD risk even beyond “normal” levels, which may vary further according to age and sex.

Most importantly, the added value of the aforementioned subclinical vascular alterations in predicting future CVD events in low- and intermediate-risk individuals with normal blood pressure levels needs to be clarified in appropriately conducted prospective studies. There is evidence suggesting that accumulation of subclinical vascular alterations may be linked with increased CVD risk, but this needs to be verified in prospective cohort studies [76]. Finally, the implementation of ambulatory blood pressure measurements may be necessary to accurately diagnose normotension and exclude individuals with masked hypertension [77], who may already present signs of HMOD and altered vascular function and morphology [65•, 78].

## Conclusion

A growing amount of prospectively obtained data supports that altered morphology and function of the micro- and microvasculature predict the development of new-onset hypertension in normotensive individuals. Specifically, arterial stiffness, increased cIMT and UAE, and altered retinal microvascular diameters predict the progression from the normotensive to the hypertensive state, whereas there is substantial lack of relevant prospective evidence for skin microvascular alterations. Although research reports suggest that such alterations precede and predispose to hypertension, through the remodeling of blood vessels and the increase in vascular resistance, conclusions regarding causality effects cannot be safely driven from available studies. Thus, identification of subtle vascular alterations in normotensive individuals could serve as a sensitive indicator of future hypertension onset and hence an increased CVD risk. It could be reasonably assumed that in these individuals, closer monitoring for the timely diagnosis of hypertension, intensification of lifestyle interventions, and perhaps early initiation of medical treatment would alleviate the global CVD burden. Appropriately designed longitudinal studies are eagerly warranted to delineate the potential role of subtle vascular alterations in primary CVD prevention.
